# Machine learning-driven analysis of feed additives and intestinal microbiota diversity in broiler chickens: Clustering of mineral profiles and predictive diet modeling

**DOI:** 10.14202/vetworld.2025.3390-3408

**Published:** 2025-11-06

**Authors:** Lyubov Sergeevna Grishina, Arthur Yurievich Zhigalov, Irina Pavlovna Bolodurina, Alexander Evgenievich Shukhman, Pavel Leonidovich Niryan, Olga Vilorievna Kvan, Elena Vladimirovna Sheida

**Affiliations:** 1 Laboratory of Artificial Intelligence and Data Analysis, Research Institute of Digital Intelligent Technologies, Orenburg State University, Pobedy Pr. 13, Orenburg 460018, Russia; 2 Department of Food Biotechnology, Orenburg State University, Pobedy Pr. 13, Orenburg 460018, Russia

**Keywords:** broilers, clustering, conditional tabular generative adversarial networks, decision tree, feed additives, gut microbiota, machine learning, mineral metabolism

## Abstract

**Background and Aim:**

The gut microbiota of broilers plays a pivotal role in nutrient absorption, immune modulation, and mineral metabolism. Feed additives can influence these microbial and physiological processes, yet their integrated effects remain insufficiently understood. This study aimed to intelligently evaluate the impact of various feed additives on the intestinal microbiota and mineral composition of broiler chickens and to develop machine learning (ML) models for clustering and classification of diet-associated mineral and microbial profiles.

**Materials and Methods:**

A total of 385 Arbor Acres broilers (7 days old) were allocated into 11 groups, including one control semi-synthetic diet (SSD), one group with a semi-synthetic deficient diet (SSDD), and nine experimental groups receiving SSDD with different additives: Probiotics (Soya-bifidum and Sporobacterin), dietary fibers (cellulose, lactulose, and chitosan), enterosorbents (enterosgel and activated carbon), and ultrafine particles (UFPs) (Cu and Fe). Microbiota composition was assessed by 16S ribosomal RNA sequencing, and body mineral composition was determined through inductively coupled plasma mass spectrometer. To overcome data scarcity, synthetic records were generated using conditional tabular generative adversarial networks. K-means and hierarchical agglomerative clustering were used for mineral profile grouping, while logistic regression, SVM, and decision tree models classified diet types.

**Results:**

Hierarchical clustering revealed six distinct mineral profile groups (Silhouette = 0.524), with SSD and SSDD forming separate clusters. Feed additives such as UFPs, chitosan, and activated carbon induced similar mineral patterns. Key differentiating biomarkers were cobalt, zinc, strontium, arsenic, and lithium (p < 0.05). The decision tree classifier achieved 74% accuracy in predicting diet types based on microbiota data. Alpha diversity analysis showed enhanced microbial richness in groups fed lactulose, enterosgel, cellulose, or activated carbon.

**Conclusion:**

ML effectively elucidated complex relationships between diet, microbiota composition, and mineral metabolism in broilers. The integration of clustering and predictive models demonstrates the feasibility of intelligent feeding systems tailored to optimize gut health and nutrient utilization. Future studies integrating multi-omics data and broader farm-level validation will strengthen precision nutrition frameworks for sustainable poultry production.

## INTRODUCTION

In recent years, rapid digitalization and the integration of artificial intelligence (AI) technologies have transformed multiple industries, including agriculture and animal husbandry [1, 2]. Modern agricultural enterprises increasingly employ AI-based solutions for crop yield forecasting, resource optimization, and animal health monitoring [3–5]. Within this transformative context, the present study emphasizes the intelligent analysis of the intestinal microbiota in broiler chickens, a critical determinant of poultry productivity and health.

Broiler performance is closely linked to feed quality, gut microbiota composition, and mineral metabolism. The gastrointestinal microbiota directly influences nutrient absorption, accumulation, and excretion. Reed *et al*. [6] demonstrated that microbiota composition affects zinc (Zn) bioavailability in hosts; a reduction in Bacillota abundance decreases short-chain fatty acid (SCFAs) production and promotes Proteobacteria proliferation. These bacteria competitively absorb Zn, exacerbating host mineral deficiencies.

Feed additives are widely used to enhance poultry health and growth [7], yet their effects on intestinal microbial diversity and community structure remain insufficiently characterized. In this regard, intelligent data analysis techniques, such as machine learning (ML) and statistical modeling, offer powerful tools to identify hidden relationships between feed additives and microbiota dynamics, enabling data-driven feed optimization for improved productivity. ML and deep learning (DL) approaches effectively model complex, non-linear microbial interactions often overlooked by traditional analytical methods [8], providing a multidimensional and integrative understanding of the microbiome’s role in metabolism and health [9].

Recent investigations have confirmed the efficacy of several additives in promoting intestinal development and nutrient utilization. Probiotics, for instance, accelerate microbiota maturation, enhance immune competence, and improve feed conversion efficiency, yielding economic benefits [10]. They stabilize intestinal flora, suppress pathogenic colonization [11], stimulate goblet cell proliferation, and enhance mucosal T-cell immunity [12]. Prebiotics such as lactulose similarly improve nutrient absorption and intestinal microbial balance, thereby supporting growth performance [13].

Given the adverse effects of antibiotic use on microbial homeostasis and interbacterial interactions [14, 15], probiotics and prebiotics are increasingly recognized as sustainable alternatives. Dietary fibers, comprising indigestible polysaccharides and lignin, reach the hindgut intact, improving feed conversion, digestion efficiency, and overall growth [16]. Enterosorbents, by contrast, mitigate xenobiotic absorption, modulate the intestinal biochemical milieu, and selectively promote beneficial microbiota while suppressing pathogens [17, 18].

Phytogenic additives, rich in bioactive polyphenols (e.g., flavonoids, eugenol, and curcumin), further support intestinal health through antibacterial, antioxidant, immunomodulatory, and anti-inflammatory mechanisms. More recently, ultrafine or nanodispersed forms of chemical elements have gained attention due to their high bioactivity, improved trace element bioavailability, and lower toxicity compared to conventional mineral forms [19]. Miroshnikova *et al*. [20] have shown that ultrafine metal particle (UFP) preparations can influence the organism’s elemental status in diverse ways; for example, UFP Cu and UFP Fe supplementation have been associated with elevated serum calcium and iron concentrations.

Despite significant progress in poultry nutrition and microbiome research, there remains a lack of integrative studies that link feed additive supplementation, intestinal microbiota composition, and mineral metabolism using intelligent computational approaches. Most previous investigations have examined these aspects independently, either evaluating microbial community shifts, nutrient absorption, or growth performance, but without systematically modeling their interrelationships. Furthermore, conventional statistical analyses often fail to capture the non-linear and multidimensional interactions that exist between gut microbial taxa, elemental balance, and dietary interventions.

Another critical gap lies in the scarcity of high-quality, comprehensive datasets for modeling microbiota–mineral interactions under various dietary conditions. The ethical and logistical limitations of animal experiments further constrain sample size and variability, restricting the use of robust predictive analytics. Consequently, the potential of ML and DL algorithms to uncover hidden patterns within such biological systems remains underutilized in poultry science. Similarly, while several studies have demonstrated the benefits of probiotics, prebiotics, and mineral additives on performance metrics, their combined impact on gut microbial diversity, mineral homeostasis, and predictive dietary classification has not been explored using synthetic data augmentation or clustering-based models.

Therefore, there is a pressing need to develop AI-driven frameworks capable of integrating microbiota sequencing, elemental composition, and feeding strategies into predictive and interpretable models. Addressing this gap would advance precision nutrition and data-driven decision-making in poultry management.

The present study aims to conduct an intelligent analysis of the intestinal microbiota and mineral metabolism in broiler chickens subjected to different feed additives by applying ML algorithms for data-driven modeling and prediction. Specifically, the study seeks to:

1. Evaluate the effects of various dietary supplements, including probiotics, prebiotics, enterosorbents, dietary fibers, and UFPs (Cu and Fe), on the intestinal microbial diversity and mineral composition of broilers.
2. Develop synthetic data using a conditional tabular generative adversarial network (CTGAN) to enhance dataset robustness and overcome experimental sample-size limitations while adhering to ethical standards in animal research.
3. Apply clustering algorithms (K-means and hierarchical agglomerative clustering [HAC]) to identify mineral profile patterns and classify groups of broilers with similar dietary responses.
4. Construct and evaluate predictive models (logistic regression, support vector machine [SVM], and decision tree) for classifying diet types and assessing the relationship between microbiota composition and nutrient deficiencies.

Through these objectives, the study aims to establish a computationally intelligent framework for understanding how feed additives modulate gut microbiota and mineral metabolism, thereby enabling the design of optimized and individualized feeding strategies that enhance broiler health, productivity, and sustainability.

## MATERIALS AND METHODS

### Ethical approval

Animal care and experimental studies were carried out in accordance with the instructions and recommendations set out in the relevant normative acts. The research followed the Model Law of the Interparliamentary Assembly of the Member States of the Commonwealth of Independent States “*On the Treatment of Animals*,” Article 20 (Resolution No. 1 of the MA of CIS Member States, May 21, 2024). Measures were taken to minimize animal suffering and reduce the number of test samples (Protocol No. 1, dated May 21, 2024).

### Study period and location

The study was conducted during May and June 2024. The experimental work was performed at the biological clinic of the Federal State Budgetary Institution of the Federal Research Center for Biological Systems and Agrotechnologies of the Russian Academy of Sciences in Orenburg (Accreditation Certificate PA.RU21PF59, December 2, 2015).

### Experimental animals

Broiler chickens of the *Arbor Acres* cross were selected for the experiment (CJSC “Orenburg Poultry Farm,” www.pfo56.ru). All birds were kept under identical environmental conditions. General diets for experimental poultry were prepared according to the recommendations of the Federal State Budgetary Scientific Institution Federal Scientific Center “*All-Russian Research and Technological Poultry Institute” (ARRTPI)* [21]. The birds were fed twice daily. Feed intake and body weight changes were monitored weekly, followed by the calculation of average daily weight gain. Anatomical (post-slaughter) carcass dissection was performed according to the ARRTPI method [22].

For the experiment, 385 broiler chickens (7 days old) were used and divided into 11 groups (n = 35) using the analog-group method. As part of the experiment, the following additives were introduced into the diets: Probiotic drugs (*Soya-bifidum* [strain *B. longum*] and *Sporobacterin* [strain *B. subtilis*]); dietary fibers (microcrystalline cellulose [E460, Hiranya Cellulse Products, India], lactulose [VTF, Moscow], and food-grade chitosan [Orison Chemicals Ltd, China]); enterosorbents (enterosgel [active ingredient: Polymethylsiloxane polyhydrate; TNK SILMA, Russia] and activated carbon [active ingredient: “Activated charcoal,” No. R N001033/01, Pharmstandard-Lexredstva, Russia]); and ultrafine particles (UFPs) (UFP copper, diameter 55 nm, Moscow, Russia; and UFP iron, diameter 90 nm, Moscow, Russia).

### Experimental design

The experimental design included the following:

- K1 (control): Semi-synthetic diet (SSD) without feed additives.
- K2: SSD deficient in trace elements semi-synthetic deficient diet (SSDD) (Fe, Mn, Cu, Zn, cobalt [Co], Mo, and Se) according to A.K. Osmanyan (Table 1).
- I experimental groups (EG): SSDD + *Soya-bifidum*.
- II EG: SSDD + *Sporobacterin*.
- III EG: SSDD + microcrystalline cellulose.
- IV EG: SSDD + lactulose.
- V EG: SSDD + food chitosan.
- VI EG: SSDD + enterosgel.
- VII EG: SSDD + activated carbon.
- VIII EG: SSDD + UFP Cu.
- IX EG: SSDD + UFP Fe.

**Table 1:** Composition of the experimental rations (g/100 g of feed).

No.	Element	Diet	

		К1 (SSD)	К2 (SSDD)
1	Casein	20	20
2	Gelatin	5	5
3	Cellulose	3	3
4	Vegetable oil	3	3
5	Choline chloride	0.2	0.2
6	Glucose	1.25	1.25
7	The rice is polished, boiled, and washed in distilled water.	61.38	61.38
8	Methionine	0.1	0.1
9	Cystine	0.2	0.2
10	CaHPO_4_*H_2_O	1.8	1.8
11	CaCO_3_	1.45	1.45
12	KH_2_PO_4_	1.013	1.013
13	KCl	0.21	0.21
14	Na_2_CO_3_	0.555	0.555
15	MnCl*4H_2_O	0.04	-
16	FeSO_4_*7H_2_O	0.05	-
17	MgSO_4_*7H_2_O	0.615	0.615
18	KJ	0.001	0.001
19	CuSO_4_*5H_2_O	0.001	-
20	ZnCl_2_	0.016	-
21	CoCl_2_	0.0002	-
22	NaMoO_4_*2H_2_O	0.0008	-
23	Na_2_SeO_3_	0.000015	-
24	Vitamin mixture *	0.052	0.052

* Vitamin mixture (mg per 100 g of feed): B1, 2.5 mg; B2, 1.5 mg; B6, 0.6 mg; B12, 0.002 mg; Ca-pantothenate, 2.0 mg; biotin, 0.06 mg; folic acid, 0.4 mg; K3, 0.5 mg; C, 25.0 mg; PP, 15.0 mg; A PER 1000 IU; D3, 360 IU; E, 0.5%. SSD=Semi-synthetic diet, SSDD=Semi-synthetic deficient diet.

The dosages used for various diets and related feed additives are shown in Table 2. Chickens were given distilled water *ad libitum*. Birds were slaughtered on day 42 under the action of etabolo ether.

**Table 2:** Dosages of the experimental diets.

No.	Abbreviation	Diet	Dosage
1	К1	Semi-synthetic diet	60 g per day for up to 28 days. 120 g. per day from day 28.
2	К2	A semi-synthetic deficient diet	60 g per day for up to 28 days. 120 g per day from day 28.
3	I EG	SSDD + soybean-bifidum	SSDD + 0.7 mL/kg of feed
4	II EG	SSDD + Sporobacterin	SSDD + 0.25 mL/kg of feed
5	III EG	SSDD + Microcrystalline Cellulose	SSDD + 0.25 g/kg feed
6	IV EG	SSDD + Lactulose	SSDD + 1 g/kg feed
7	V EG	SSDD + food chitosan	SSDD + 0.5 g/kg of feed
8	VI EG	SSDD + Enterosgel	SSDD + 6.0 g/kg of feed
9	VII EG	SSDD + activated carbon	SSDD + 3.0 g/kg of feed
10	VIII EG	SSDD + UFP Cu	SSDD + 1.7 mL/kg of feed
11	IX EG	SSDD + UFP Fe	SSDD + 17.0 mL/kg of feed

SSDD = Semi-synthetic deficient diet, EG = Experimental groups.

### Sample collection and laboratory analyses

During slaughter, composite samples of muscle, skin, internal organs (tissues of the gastrointestinal tract, heart, lungs, liver, kidneys, spleen, and gonads), feathers, bone tissue with central nervous system, and internal fat were collected [23]. Laboratory analyses determined the moisture content of feed (Government standard [GOST] 13586.5–93; GOST 29143–91), crude fiber (GOST 13496.2–84), protein (GOST 10846–74), and ash content (GOST 10847–74).

Before slaughter, broilers were deprived of water for 4–6 h and feed for 12 h. Birds were weighed before and after slaughter, and the individual tissues and organs were also weighed. During anatomical dissection (GOST 13496.0–70), average samples of muscle, bone, skin, internal organs, and fat were prepared for each bird. Homogenization was performed using a laboratory mill, followed by drying at ≤70°C. Samples were stored in sealed glass containers.

The indoor environment complied with ARRTPI recommendations. Growth and development were assessed weekly through individual weighing and calculation of average daily gain. All birds were reared under identical housing and feeding conditions.

Collected samples were used to determine the chemical and elemental composition of poultry tissues. The chemical composition of droppings, feed, and body tissues was analyzed according to GOST 31640–2012, GOST 32044.1–2012, GOST 13496.15–2016, GOST 33319–2015, GOST 23042–2015, GOST 25011–2017, and GOST R 31727–2012. Analyses were conducted at the Center for Collective Use of Biological Systems and Agrotechnologies of the Russian Academy of Sciences (https://ckp-rf.ru/ckp/77384/). The elemental composition of biosubstrates and compound feeds was determined using an Agilent 7900 inductively coupled plasma mass spectrometer with a 1260 Infinity II BIO-Inert high-performance liquid chromatography system.

### DNA extraction and sequencing

Samples of intestinal contents were collected aseptically into sterile Eppendorf-type microtubes (“Nuova Aptaca S.R.L.,” Italy). DNA was extracted using a modified lysis procedure. Samples were incubated at +37°C for 30 min in 300 μL of sterile lysis buffer (20 mM ethylenediaminetetraacetic acid, 1,400 mM NaCl, 100 mM Tris-HCl, pH 7.5) containing 50 μL lysozyme solution (100 mg/mL). Then, 10 μL of proteinase K (10 mg/mL; “Thermo Fisher Scientific, Inc.,” USA) and sodium dodecyl sulfate (1%) were added, followed by incubation for 30 min at +60°C.

Microbial biodiversity was analyzed by next-generation sequencing using a *MiSeq* sequencer (Illumina Inc., USA) with theMiSeq Reagent Kit v3 (600 cycles, Iluster density 1.300 K/mm², Illumina Inc.) at the Center for Collective Use of Scientific Equipment “*Persistence of Microorganisms*.” DNA libraries were prepared according to the *Illumina* protocol using primers S-D-Bact-0341-b-S-17 and S-D-Bact-0785-a-A-21 for the V3–V4 region of the 16S ribosomal RNA (*16S rRNA*) gene. Sequencing was performed using the *MiSeq Reagent Kit V3 PE600* platform.

Operational taxonomic units (OTUs) were classified using the *VAMPS* tool and the *RDP* database (http://rdp.cme.edu). Selected OTUs were aligned using the Basic Local Alignment Search Tool algorithm with the *nr/nt* database (National Center for Biotechnology Information) and *SILVA* ribosomal *RNA* gene sequences.

### Sequencing and bioinformatics

A total of 814,981 reads were obtained for prokaryotes (*16S rRNA* gene), of which 567,814 passed quality filters. Data from 33 samples (average sequencing depth: 21,318 reads per sample; average read length: 251 bp) were used. For alpha diversity analysis, sequences were rarefied to a minimum depth of 15,814 reads per sample.

Bioinformatic processing used the PEAR v0.9.8 program (Exelixis Lab, Germany). Filtering, de-duplication, chimera removal, clustering, and contamination filtering were performed inUSEARCH v11.0.667 (Edgar R.C., 2010, USA) using *fastq_filter, derep_prefix*, and *cluster_otus* algorithms. Results were processed inMicrosoft Excel 2021 (Microsoft Office, Washington, USA).

### Dataset description

During the experiment, data on body mineral composition (BMC), intestinal microbiota, and weekly body weights were collected to establish physicochemical relationships and construct predictive machine-learning models.

The mineral dataset contained 25 chemical elements grouped as follows: Alkali metals (Li, Na, and K); alkaline earth metals (Mg and Ca); transition metals (V, Cr, Mn, Fe, Co, Ni, Cu, and Zn); post-transition metals (Sn and Pb); semi-metals (Al, Si, and As); non-metals (P, Se, and I); and heavy metals (Hg and Cd).

The microbiota dataset included hierarchical levels: Phyla, classes, orders, families, and species. The intestinal microbiota was dominated by *Actinobacteria, Bacteroidetes, Firmicutes*, and *Proteobacteria*, which were divided into 11 classes: *Actinobacteria, Coriobacteriia, Bacteroidia, Bacilli, Clostridia, Erysipelotrichia, Alphaproteobacteria, Betaproteobacteria, Deltaproteobacteria, Dothideomycetes*, and *Gammaproteobacteria*. Table 3 presents the division of microbiota taxa into orders and their functions in the broiler intestine. The dataset also includes family-level (38 total) and species-level (108 total) details.

**Table 3:** Characteristics of intestinal microbiota bacteria at the order level.

No.	Order	Characteristic
1	*Micrococcales*	Moderate effect, some synthesis of vitamins, minimal effect on chicken growth
2	*Mycobacteriales*	Rarely found in the digestive tract of birds, potentially harmful
3	*Eggerthellales*	They modulate bile acid metabolism and improve fat absorption
4	*Bacteroidales*	The main producers of short-chain fatty acids (acetate and propionate): increases the intestinal mucosa’s energy exchange and improves feed conversion
5	*Bacillales*	Bacillus and Enterococcus improve villi growth and immunity, whereas Staphylococcus and Listeria are pathogenic to dysbiosis
6	*Lactobacillales*	They lower pH, suppress pathogens, and stimulate digestion and weight growth
7	*Clostridiales*	Nourish the epithelium, stimulate the immune system/*Clostridium* *perfringens* cause necrotizing enteritis
8	*Erysipelotrichales*	Improved feed conversion may cause inflammation
9	*Kiloniellales*	They do not participate in feed fermentation
10	*Rhizobiales*	Methanol/magnesium is metabolized; however, its role in the digestive tract of birds is minimal
11	*Burkholderiales*	The effect of amino acid and organic acid exchange on productivity is insignificant
12	*Bdellovibrionales*	Can control pathogenic bacteria
13	*Enterobacteriales*	Non-pathogenic *Escherichia coli* produce vitamin K (neutral + ), whereas pathogenic *Salmonella/Klebsiella* cause enteritis and reduce productivity.
14	*Pseudomonadales*	They can absorb a wide range of organics; however, with dysbiosis, they cause inflammation
15	*Xanthomonadales*	Plant phytopathogens are rare in the bird digestive tract

**Figure 1:**
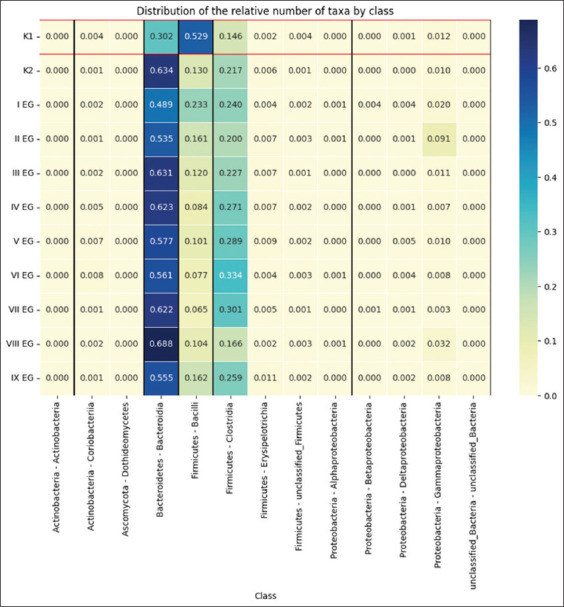
The spread of the relative number of taxa by class.

Microbiota data were normalized using the Normalizer (norm = “11”) method to preserve relative proportions and eliminate scale effects. This L1 normalization ensures that the sum of absolute values for each vector equals one. Figure 1 presents the relative abundance of taxa by phylum.

Across all groups, the predominant phyla were *Bacteroidetes* and *Firmicutes*. Groups II, III, IV, VII, and VIII showed predominance of *Bacteroidetes*, and in group VIII, their number was twice as high as in additive-free groups. *Bacteroidia* was the most abundant class in groups II, III, IV, and VIII. *Ascomycota* appeared only in group III. In general, the classes *Bacteroidia, Bacilli*, and *Clostridia* dominated all diets, while *Dothideomycetes* represented the smallest fraction. Under SSD diet, *Bacilli* predominated, whereas in group VI, *Clostridia* were dominant.

### Microbiota-mineral interaction mechanisms

The intestinal microbiota influences mineral metabolism through several biological mechanisms. Microorganisms of the major phyla (*Actinobacteria, Bacteroidetes, Firmicutes*, and *Proteobacteria*) affect mineral assimilation by enzymatic decomposition of feed components and synthesis of biologically active substances.

A key pathway involves fermentation of complex carbohydrates and fibers, producing SCFAs such as acetate, propionate, and butyrate. SCFAs enhance intestinal absorption of minerals (Ca, Mg, and Fe) by lowering pH and improving mineral salt solubility. *Bacilli* and *Clostridia* participate in sulfate-reduction and phosphate-metabolic pathways, regulating sulfur and phosphorus balance. *Deltaproteobacteria* activity is linked with reduction processes affecting trace-element availability.

Certain bacteria synthesize vitamins (e.g., Vitamin K) essential for bone mineralization and produce substances that facilitate mineral-ion transport across intestinal epithelia. Microbial metabolites may also regulate gene expression associated with minerotropism.

Differences in microbiota composition (e.g., dominance of *Bacteroidetes* and *Clostridia* in specific groups) correspond to variations in mineral metabolism, indicating an intimate link between microbial ecology and mineral assimilation/excretion.

Some EG exhibited similar microbiota profiles; for example, lactulose supplementation yielded comparable ecological niches, while others displayed distinct diversity patterns, reflecting ecological variability. Overall, the dataset describing BMC and intestinal microbiota supports investigation of complex dependencies and facilitates machine-learning modeling of additive effects.

### Synthetic data generation

Conducting animal-related experiments requires considerable time and a suitable material base. The number of records studied for each group (n = 35) exceeds the threshold of 30 observations, at which the Central Limit Theorem provides an approximation to the normal distribution, allowing the correct application of parametric tests and ML models. However, the data describe complex relationships among components within a small sample size, which can lead to biased results. Synthetic data can help model these relationships, allowing expansion of the original dataset and assessment of how different combinations affect broiler condition and productivity. In addition, the use of synthetic data makes it possible to minimize the number of experiments on animals, in accordance with modern ethical standards.

Synthetic data can be generated using several approaches, including modeling based on statistical distributions (for example, normal, binomial, or Poisson), generative adversarial networks (GAN), variational autoencoders, simulation methods (agent-based modeling or systems of equations), and others [24–26].

As part of this research, a CTGAN-based method was implemented, which relies on concepts used in GAN but is adapted to work with continuous, categorical, and temporal data [27]. This approach allows generation of synthetic data that preserve the statistical properties of the original dataset, including relationships between variables. The method uses the GAN architecture consisting of a generator that creates synthetic data and a discriminator that evaluates its realism in comparison with real data.

An additional n = 165 synthetic records were generated for datasets on the BMC and intestinal microbiota of broilers using the CTGANSynthesizer model (https://github.com/sdv-dev). The Column Shapes Score = 71.29% indicates moderate correspondence between synthetic and original column-shape distributions. The Column Pair Trends Score = 82.45% shows that the synthetic data successfully reproduce inter-variable trends and dependencies. The Overall Score (Average) = 76.87% confirms that the generated dataset is of satisfactory quality and can be used to train machine-learning models.

**Figure 2:**
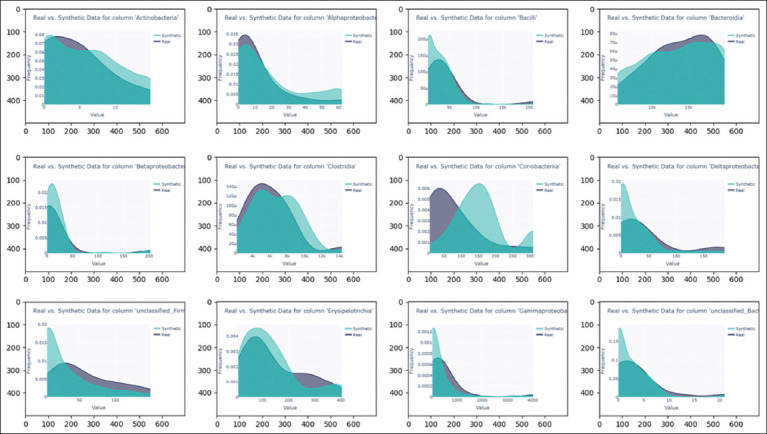
Kernel-density-estimation graphs of real and synthetic taxon data by class.

**Figure 3:**
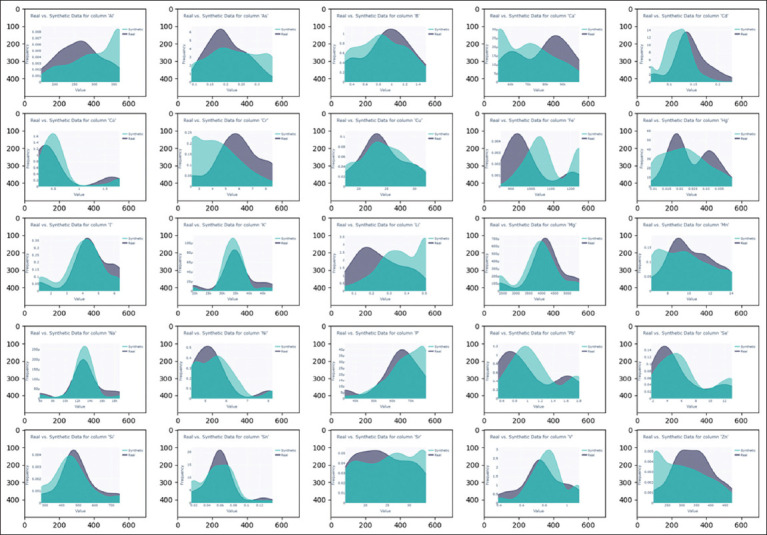
Kernel-density-estimation graphs of the mineral composition data of real and synthetic broiler bodies.

Kernel-density-estimation graphs of real and synthetic taxon data by class and body-mineral composition are shown in Figures 2 and 3, respectively. More than half of the bacterial classes in the synthetic data have distributions that are close to the real ones, whereas *Alphaproteobacteria, Erysipelotrichia*, and *unclassified Bacteria* display broader distributions. All chemical elements of the body’s mineral composition in the synthetic dataset have distributions close to those observed in the real data.

The results demonstrate that CTGANSynthesizer successfully generates synthetic data that retain many of the statistical and biological characteristics of the original dataset. However, due to inherent limitations of data-generation methods, synthetic data were used only for the training dataset, while machine-learning models were tested exclusively on real data.

### Correlation analysis of data

**Figure 4:**
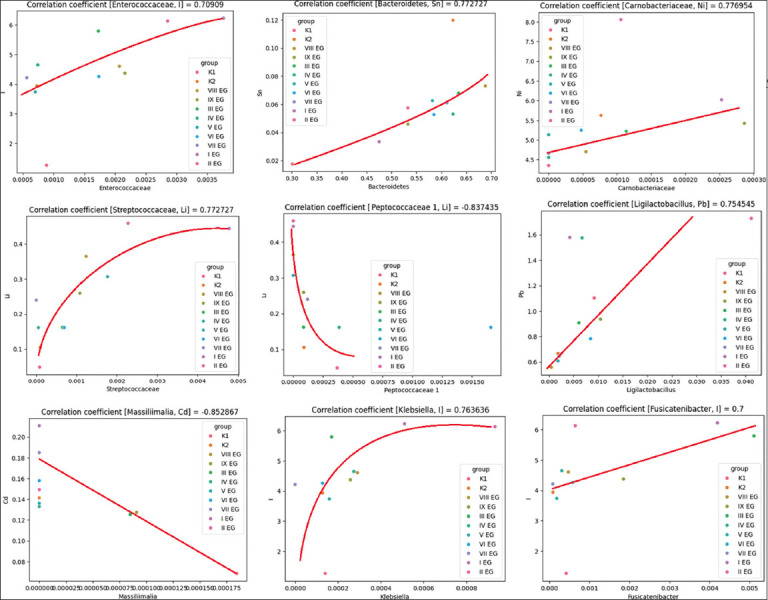
Graphs of the distribution of taxa by elements of the mineral composition of the body.

A correlation analysis was conducted to assess the relationship between the abundance of microbial taxa in the avian gut microbiota and the concentrations of specific chemical elements in the body’s mineral composition. The Spearman rank correlation coefficient, a non-parametric statistical method robust to deviations from normality and the presence of outliers, was employed for this purpose, making it particularly suitable for biological data. Visualization of significant associations, defined as those with a Spearman correlation coefficient exceeding 0.7, is presented in Figure 4.

The highest positive correlation was observed in the pair [*Roseburia, Si*] = 0.82, which may indicate the importance of this taxon in the context of silicon metabolism. Moderately positive correlations were identified between pairs (*Lachnospiraceae incertae sedis, B*) = 0.7927, (*Flavonifractor, B*) = 0.7727, and *(Escherichia_Shigella, Ca*) = 0.8049. Other taxa, such as *Lachnospiraceae incertae sedis, Klebsiella*, and *Lactococcus*, also showed moderate positive correlations with elements, such as lithium (Li) and nickel, suggesting possible roles in the metabolism of these minerals.

Strong negative correlations were observed between (*Massiliimalia, Cd*) = –0.8529 and (*Massiliimalia, Mn*) = –0.8108, indicating that the presence of *Massiliimalia i*s associated with low cadmium and manganese concentrations. A significant negative correlation was also found for (*Hydrogeniiclostridium, Cr*) = –0.7636. In addition, taxa such as *Faecalibacterium* and *Petroclostridium* showed negative correlations with selenium, suggesting their potential influence on selenium levels in the body.

Thus, the correlation analysis revealed both positive and negative associations between gut-microbiota taxonomic diversity and the concentrations of specific elements in the body’s mineral composition. Strong positive correlations may reflect synergistic physiological or ecological interactions, possibly indicating element-dependent modulation of microbial communities, while negative correlations may suggest competitive or inhibitory relationships that merit further investigation.

### Data diversity analysis

Alpha diversity of the microbiota, a widely used ecological and microbiological metric for assessing species richness and evenness within an ecosystem, was analyzed to characterize the complexity and structure of the gut microbiota in the studied broilers.

Alpha diversity describes the diversity within a single community or sample. Several indexes are available for its calculation, each reflecting different aspects of diversity and sensitivity to ecological or sampling factors. The principal purpose of alpha-diversity calculation is to compare the total number of species within samples and assess the evenness of their distribution.

The main methods for assessing alpha diversity include the Shannon index, Simpson index, and species richness. In this study, the Simpson Diversity Index was chosen because it focuses on species richness, allows comparisons between systems, and is less sensitive to sample size than the Shannon index.

The Simpson index is defined as:


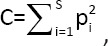


**Figure 5:**
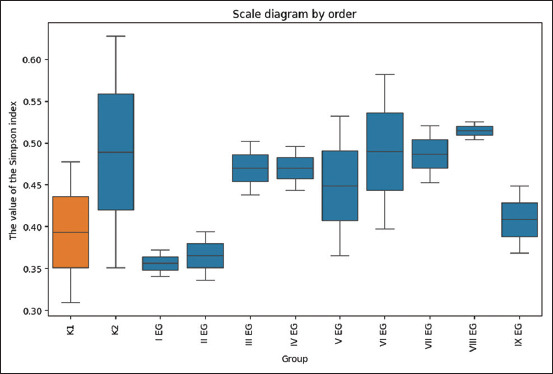
The values of the Simpson index for the aggregation level are in order.

Where *p_i_* is the proportion of individuals belonging to species *I* among all individuals, and *S* is the number of species observed. The Simpson index measures the relationship between intraspecific and interspecific interactions. Its value ranges from 0 to 1, representing minimal to infinite variety, respectively. Figure 5 presents a diagram showing the Simpson-index range for intestinal microbiota at the order level.

For the SSD, which characterizes a richer mineral composition, the lower limit of the Simpson index was 0.32, indicating less minimal diversity compared with SSDD, while the upper limit was 0.48, suggesting that a richer diet may promote dominance of certain species and thus limit overall variety. The SSDD group displayed a wider range of diversity, indicating that under dietary deficiency, species interact more actively and adapt to limited resources.

Comparative evaluation of alpha diversity among supplement groups revealed that:

1. EG I, II, and IX exhibited lower minimum values (<0.37), suggesting a reduced variety of species when these additives were used, possibly limiting microbiota diversity.
2. EG III, IV, V, VI, VII, and VIII showed higher lower-limit values of alpha diversity, suggesting that these additives positively influence the maintenance of microbial biodiversity.

### Clustering methods for BMC

To identify similar profiles of BMC among broilers fed different diets (SSD, SSDD, or SSDD + additives), unsupervised machine-learning methods were applied: K-means and HAC.

#### K-means

K-means is a clustering algorithm that divides a dataset into *k* specified clusters [28]. It iteratively assigns data points to clusters based on similarity and updates centroid positions according to the mean values of each cluster until convergence (no change in centroids). The algorithm was implemented in Python 3.12 using the Scikit-learn 1.6.1 library.

#### HAC

HAC constructs a hierarchical cluster structure [29]. The algorithm operates on a “bottom-up” basis, starting with each object as a separate cluster and progressively merging them into larger clusters. Unlike K-means, HAC does not require a predefined number of clusters and enables visualization as a dendrogram. The method was implemented using the agglomerative clustering object from Scikit-learn and dendrogram visualization from the Scipy 1.15.2 library.

#### Clustering quality metrics

The following metrics were used to evaluate clustering quality [30, 31]:

- Silhouette score: Considers both intra- and inter-cluster density (–1 to 1; values near 1 indicate good separation).
- Calinski-Harabasz index: Measures the ratio of between- to within-cluster variance (higher values = better clustering).
- Davies-Bouldin index: Represents the average ratio of inter-cluster distance to intra-cluster compactness (lower values = better clustering).

Each metric offers distinct strengths; therefore, they were used jointly for a comprehensive evaluation. Implementation of these clustering algorithms to identify body-mineral-composition profiles of broilers under different diets is described in the Results section.

### Forecasting of deficient diets

Diet composition directly affects both the mineral content of the body and the structure of the intestinal microbiota. The most commonly used feed formulation is the SSD, comprising natural ingredients (cereals and plant materials) and synthetic components (amino acids, vitamins, and minerals). This formulation provides optimal conditions for gut-microbiota development and ensures adequate intake of essential nutrients and trace elements.

In this context, the development of a predictive model to classify diet type, specifically semi-synthetic or nutrient-deficient feed, based on intestinal-microbiota data from broilers, while accounting for nutrient-deficiency compensation by dietary additives, represents a timely and scientifically relevant objective.

To address this task, the microbiota data were divided into two groups:

- SSD: Records corresponding to SSD
- Non-SSD: Records representing all other diets (10 types in total, including the deficient and nine supplemented diets with probiotics, fibers, enterosorbents, and UFPs).

The dataset was unbalanced, with a class ratio of 1:10 (SSD: Non-SSD). To control overfitting, fivefold cross-validation was applied. Data were split into training (70%), validation (10%), and testing (20%) subsets.

Model performance was evaluated using a confusion matrix and the accuracy metric, which represents the proportion of correctly predicted cases among all model forecasts.

The following machine-learning models were implemented to solve the binary-classification problem of identifying SSD: Logistic Regression, SVM, and Decision Tree. These relatively lightweight models, with limited trainable parameters, perform effectively on small datasets without overfitting. Key hyperparameters optimized during training are summarized in Table 4.

**Table 4:** Hyperparameters of the machine learning models used to determine diet.

No.	Model	Parameter	Description
1	Logistic regression	C	Regularization coefficient
		penalty	The penalty rate
2	SVM	C	Regularization coefficient
		kernel	The kernel of the proposed model
		degree	Degree of polynomial
3	Decision tree	max_depth	Maximum decision tree depth
		criterion	The tree branching criterion

SVM = Support vector machine.

To reduce class-imbalance effects during model training, a weighted loss function was applied, increasing the error penalty for the minority class. In addition, principal component analysis (PCA) was used to compress the feature space and enhance classification accuracy. The number of retained components ranged from 2 to the total number of initial features.

Implementation details of the classification algorithms and the identification of broilers on deficient diets using intestinal-microbiota data are presented in the Results section.

## RESULTS

### Analysis of clustering data results

K-means and agglomerative clustering algorithms were implemented to identify similar profiles of the BMC of broilers consuming different diets. The best hyperparameters were selected based on a series of experimental runs, and clustering quality was evaluated using the previously described metrics. The detailed results are presented in Table 5.

**Table 5:** Clustering data results on the mineral composition of broilers.

No.	Model	Hyperparameters	Silhouette	Kalinski-Harabas	Davis-boldin
1	K-means	Euclidean, k = 2	0.429042	32.447942	0.789693
		Euclidean, k = 4	0.429173	46.153924	0.965046
		Euclidean, k = 6	0.455775	40.727667	1.018787
2	Hierarchical (average)	Euclidean, k = 2	0.445899	21.849180	0.499230
		Euclidean, k = 4	0.429173	46.153924	0.965046
		Euclidean, k = 6	**0.524312**	**46.937840**	0.716781
		Manhattan, k = 2	0.445899	21.849180	0.499230
		Manhattan, k = 4	0.429173	46.153924	0.965046
		Manhattan, k = 6	0.489519	45.080141	0.797523
		Chebysheve, k = 2	0.445899	21.849180	0.499230
		Chebysheve, k = 4	0.320102	31.933266	0.908929
		Chebysheve, k = 5	0.487386	42.491278	0.960623
		Minkowski (p = 1.5), k = 2	0.445899	21.849180	0.499230
		Minkowski (P = 1.5), K = 4	0.429173	46.153924	0.965046
		Minkowski (p = 1.5), k = 6	0.524312	46.937840	0.716781

The Silhouette score and the Kalinski-Harabas index are highlighted in bold, which correspond to the most effective configuration of the clustering model parameters.

#### Selection of optimal clustering parameters

According to the experimental results, the HAC algorithm with the Euclidean distance and a cluster number of 6 (k = 6) yielded the best results. Based on the silhouette coefficient (0.5243) and the Calinski–Harabasz index (46.9378), this configuration achieved the best balance of inter- and intra-cluster separation (bold values in Table 5).

**Figure 6:**
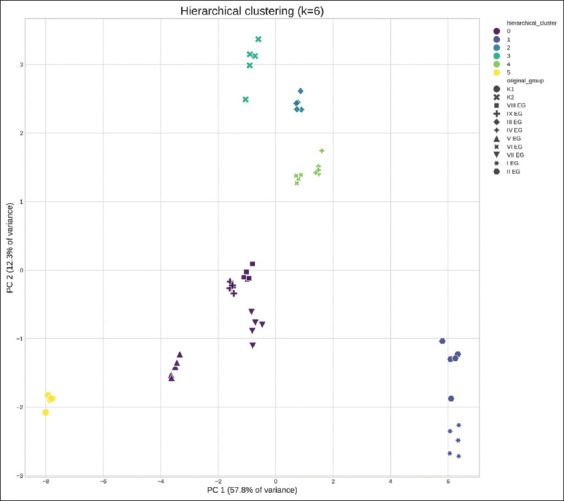
The principal component analysis method for visualizing hierarchical clustering.

The Davies-Bouldin index produced less informative results, likely because it does not effectively account for the complex structure and irregular shapes of the clusters. Figure 6 illustrates the clustering visualization derived using the PCA method, while Table 6 describes the six resulting clusters according to dietary intake.

**Table 6:** Splitting food groups into clusters.

Cluster number	Diet
1	V, VII, VIII, IX
2	I, II
3	III
4	K2
5	IV, VI
6	K1

#### Cluster composition and dietary grouping

The analysis revealed that the semi-synthetic and deficient diets formed distinct and well-separated clusters. The most widespread was Cluster 1, which represented a mineral profile common to broilers receiving UFPs additives, chitosan dietary fiber, and the enterosorbent activated carbon.

The next major cluster corresponded to broilers that received probiotic feed additives (*Soya-bifidum* and *Sporobacterin*). Another closely related cluster comprised birds supplemented with Enterosgel and lactulose, both of which produced a similar mineral composition profile.

#### Statistical validation of clustering

Statistical analysis confirmed the reliability of the six-cluster solution. Cluster formation was driven primarily by significant differences in the concentrations of Co, Zn, strontium (Sr), arsenic (As), and Li (analysis of variance [ANOVA], p < 0.05 for all).

The most pronounced differences were observed for Co and Zn, which distinctly characterized Cluster 2 (I EG and II EG). This cluster showed an abnormally high Co concentration (mean = 1.633), whereas Co levels were below detection limits in Cluster 6 (K1 diet). Similarly, the Zn concentration in Cluster 2 averaged 457.0 units, more than twice that of the control group (mean = 210.8 units). These differences were statistically significant (Tukey test, p < 0.01 vs. control).

Cluster 5 (VI EG and IV EG) had the highest Sr content (mean = 31.8 units), which was 1.65× higher than Cluster 1 (mean = 19.3 units, *p* = 0.027) and almost twice the control value (mean = 16.6 units). In Cluster 5, the As content (average = 0.284 units) also exceeded that of the control group (average = 0.130 units) by more than two-fold.

The Li concentration differed significantly between clusters: Cluster 2 had an average of 0.459 units, more than 9 times higher than Cluster 6 (mean = 0.049 units, p < 0.015).

No statistically significant differences were observed between clusters for most other minerals, including Na, Mg, Fe, and Cu. The exception was calcium (Ca), for which ANOVA indicated overall significance (p = 0.0479), although Tukey’s *post hoc* test did not identify specific pairwise differences. This pattern may suggest subtle variations distributed across multiple clusters that individually do not reach statistical significance.

#### Key mineral biomarkers defining clusters

Based on the statistical and clustering analyses, Co, Zn, Sr, As, and Li were identified as key biomarkers defining the separation of broiler groups by mineral composition. These elements collectively explain the physiological divergence observed among the dietary treatments.

## Analysis of diet classification results

### Model training and hyperparameter optimization

To develop predictive models for determining the type of diet (semi-synthetic or deficient feed) based on intestinal-microbiota data, a series of experiments was conducted. The objective was to assess whether machine-learning algorithms could accurately classify diet type while accounting for additive-based compensation of nutrient deficiencies.

Model training included hyperparameter optimization, and the number of principal components used for feature-space compression was treated as an additional hyperparameter. The model-performance results are summarized in Table 7.

**Table 7:** Results of training classification models based on microbiota data.

No.	Model	Parameter	Accuracy	Precision	Recall	F1-score
1	Logistic regression	“C” = 0.0001, “penalty” = “l2”	0.62	0.58	0.65	0.61
		“C”: 1e-06, “penalty” = “l2,” components=3	0.61	0.56	0.63	0.59
2	SVM	“C” = 10000.0, “degree” = 2, “kernel” = “rbf,”	0.58	0.53	0.58	0.55
		“C” = 1000.0, “degree” = 2, “kernel” = “rbf,” components=7	0.61	0.55	0.60	0.57
3	Decision tree	“criterion” = “log_loss,” “max_depth” = 5	0.64	0.61	0.66	0.63
		“criterion” = “gini,” “max_depth” = 5 components=3	**0.74**	**0.70**	**0.75**	**0.72**

The most effective metrics of the decision tree model are highlighted in bold and correspond to the selected parameters. SVM = Support vector machine.

### Model performance and comparative evaluation

**Figure 7:**
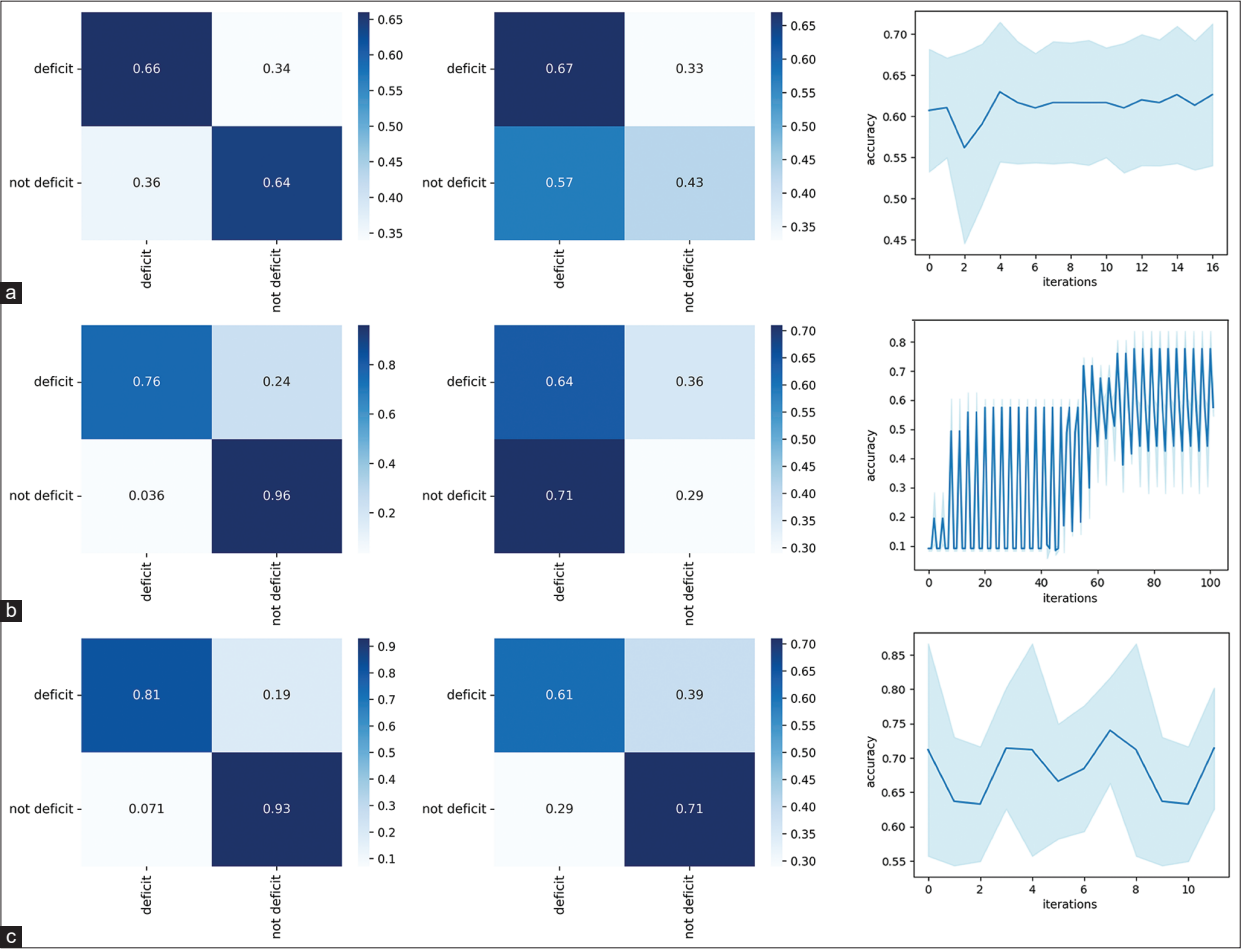
Error matrices for determining a semi-synthetic microbiota-based diet: (a) Logistic regression, (b) support vector machine, and (c) decision tree.

Error matrices for each optimal model configuration are visualized in Figure 7, corresponding to the best combination of algorithm parameters. Among all models tested, the Decision Tree classifier achieved the highest prediction accuracy, approximately 74%, outperforming both Logistic Regression and SVM models (bold values in Table 7).

This result suggests that the Decision Tree model provided the best balance of interpretability, robustness to feature interactions, and sensitivity to microbiota-driven variation in dietary classification. The findings demonstrate that microbial composition data can be effectively utilized for predicting diet type, especially when supplemented with optimized preprocessing and dimensionality-reduction strategies.

## DISCUSSION

### General overview of ML applications

The results of this study demonstrate the potential of unsupervised and supervised ML methods in analyzing BMC profiles and classifying broiler diet types based on intestinal microbiota data. The combined use of synthetic data generation, clustering, correlation analysis, and classification models provided an integrated framework for understanding diet–microbiota–mineral interactions in broilers.

### Synthetic data generation and evaluation

The generation of additional synthetic data on the BMC and intestinal microbiota of broilers using the CTGAN Synthesizer represents a significant step forward in analytical modeling. The synthetic data accurately reflected the trends and relationships between the variables (Column Pair Trends Score = 82.45%), and the overall accuracy (Overall Score = 76.87%) confirmed that the generated dataset was suitable for training ML models.

It is worth noting that the use of synthetic data has its limitations: While these data show distributions close to real ones, they cannot fully reproduce the biological variability inherent in living systems [32]. Consequently, synthetic data were used exclusively during the training stage, whereas real data were employed for validation and model evaluation. This hybrid strategy ensured both ethical compliance and analytical robustness, reducing the need for additional animal experimentation while maintaining data reliability.

### Clustering and identification of mineral-based diet groups

Clustering methods, such as HAC, proved effective in identifying six distinct groups of broilers consuming different diets (Silhouette Score ≈ 0.524; Calinski–Harabasz Index ≈ 46.937). The semi-synthetic and deficient diets formed separate clusters, suggesting that these two dietary regimes significantly affect the mineral status of birds. This observation aligns with previous reports emphasizing the critical role of balanced nutrition in maintaining micronutrient homeostasis and overall physiological health [33].

Statistical analysis (ANOVA and Tukey test) confirmed that Co, Zn, Sr, As, and Li are key biomarkers determining the clustering pattern based on BMC. Among the identified clusters, the largest group comprised diets supplemented with UFPs, chitosan dietary fibers, and activated carbon (enterosorbent). These supplements regulate intestinal microflora and improve nutrient absorption due to their prebiotic and detoxifying properties [34].

The similarity of mineral composition profiles in this group supports the hypothesis that such additives can partially compensate for nutrient deficiencies, thereby influencing mineral metabolism and availability. For example, in the case of UFP Cu, competition for common transporters of divalent metals (Cu, Zn, Pb, and Cd) in the intestine may occur. UFPs can enter intestinal cells without requiring transport proteins, due to their high penetrating ability [35]. Hence, transport systems that usually mediate Cu uptake may be repurposed for other divalent analogs in the presence of UFPs, whereas they likely perform Cu transport in diets without such additives [36].

### Role of probiotics and enterosorbents in mineral regulation

Another notable cluster corresponded to diets containing probiotic strains (*Bifidobacterium longum* and *Bacillus subtilis*), indicating that probiotics play a crucial role in shaping the mineral profile. Probiotic supplementation in monogastric diets affects the exchange of chemical elements by modulating bacterial activity and selective nutrient losses during enteral homeostasis [37, 38].

These findings are consistent with a study by El-Hack *et al*. [39] showing that probiotics enhance mineral bioavailability by modifying intestinal pH and producing organic acids that increase the solubility and absorption of Ca, magnesium, and iron. Similarly, another cluster containing enterosgel and lactulose suggests that enterosorbents and prebiotics can influence mineral dynamics by supporting beneficial bacterial populations that facilitate mineral absorption [40].

Collectively, these results indicate that feed additives such as probiotics, prebiotics, and enterosorbents can be used as complex premixes, combining mineral and biologically active substances to improve broiler productivity and gut health. Among these, probiotic strains remain the priority candidates due to their consistent effects on microbiota stability and mineral balance.

### Predictive modeling of diet classification

The Decision Tree model achieved an accuracy of approximately 74%, demonstrating promising potential for classifying diet types based on intestinal microbiota composition. This level of accuracy is particularly encouraging considering the high dimensionality and heterogeneity of microbiome data.

ML models applied to microbiota datasets often face challenges due to sparsity and feature redundancy; however, the application of dimensionality reduction methods such as PCA improves both interpretability and computational efficiency [41]. Similar study by Wang, X. and Liu [42] using tree-based classifiers to predict host phenotypes from microbiota data have reported comparable accuracies, further supporting the validity of the current approach.

Although the overall accuracy was moderate, it is important to note that the classification task involved distinguishing not only between semi-synthetic and deficient diets but also among multiple additive-enriched diets, each influencing the microbiota in unique ways. Future improvements could incorporate additional biological layers such as gene expression, metabolomic or proteomic data to enhance predictive power and biological interpretability [43].

### Correlation analysis between microbiota and mineral composition

The correlation analysis between intestinal microbiota taxa and chemical elements in the body’s mineral composition provided valuable insight into microbe–mineral interactions. Using the Spearman correlation coefficient, which accommodates the non-parametric nature of biological data, both positive and negative correlations were identified.

Strong positive correlations were observed for the pairs (*Roseburia*, Si) (r = 0.82) and (*Escherichia/Shigella*, Ca) (r = 0.804), suggesting that these taxa may play significant roles in the metabolism of silicon and Ca, respectively. Conversely, strong negative correlations were recorded for (*Massiliimalia*, Cd) (r = –0.85) and (*Massiliimalia*, Mn) (r = –0.81), indicating potential species-level competition or inhibitory interactions between these microbes and metal elements. These results contribute to the growing evidence that specific microbial taxa are closely associated with host mineral uptake and regulation.

## CONCLUSION

The present study demonstrated the successful integration of ML techniques with biological and mineral data to analyze and predict the effects of dietary interventions on broiler health. The combined use of unsupervised clustering, supervised classification, and synthetic data generation provided a holistic framework for understanding the complex interplay between diet composition, intestinal microbiota, and BMC.

The CTGAN-based synthetic data generation effectively expanded the dataset while maintaining structural integrity (Overall Score = 76.87%; Column Pair Trends Score = 82.45%), facilitating ethical and statistically balanced modeling. HAC identified six distinct broiler groups (Silhouette Score ≈ 0.524; Calinski–Harabasz Index ≈ 46.94), primarily differentiated by Co, Zn, Sr, As, and Li concentrations. These minerals were confirmed as key biomarkers governing the mineral-status divergence among dietary regimes. The Decision Tree classifier achieved an accuracy of approximately 74% in predicting diet types based on microbiota composition, validating the applicability of ML models to complex biological datasets. Correlation analyses further revealed strong associations between taxa such as *Roseburia*–Si and *Escherichia/Shigella*–Ca (positive), and *Massiliimalia*–Cd and *Massiliimalia*–Mn (negative), highlighting microbial–mineral interdependencies.

These findings underscore the feasibility of applying AI-driven analytics in precision poultry nutrition, enabling targeted feed optimization, micronutrient balancing, and improved productivity. The clustering-based identification of diets containing probiotics, UFPs, and enterosorbents provides empirical support for their inclusion as functional additives to enhance mineral bioavailability and gut health.

The study’s main strength lies in its multi-level integration of microbiological, mineral, and computational analyses. However, the cross-sectional design and absence of temporal microbiome data limited insights into dynamic microbial responses. Future research should integrate longitudinal multi-omics datasets (metagenomics, transcriptomics, and metabolomics) to refine predictive accuracy and reveal causal mechanisms in diet–microbiota–mineral interactions.

This study establishes a novel methodological foundation for data-driven poultry nutrition, demonstrating that ML-based modeling can effectively bridge biological complexity and practical feeding strategies, paving the way for sustainable, intelligent, and ethically optimized poultry production systems.

## DATA AVAILABILITY

The supplementary data can be made available from the corresponding author upon request.

## AUTHORS’ CONTRIBUTIONS

LSG, AYZ, IPB, AES, PLN, OVK, and EVS: Contributed to the conception and design of the study. LSG: Conceptualization, data analysis and interpretation, and manuscript drafting, editing, and revision. AYZ: Conducted computational experiments and trained machine learning models. IPB: Supervised the study and edited the manuscript. AES: Critical review and manuscript drafting. PLN: Conducted computational experiments and selected intelligent model parameters. OVK: Data and sample collection, laboratory tests, data analysis, and interpretation. EVS: Statistical data analysis and manuscript drafting, and editing. All authors have read and approved the final version of the manuscript.
